# Data on multi-actor parameter design tasks by engineering students with variable problem size, coupling, and team size

**DOI:** 10.1016/j.dib.2018.08.162

**Published:** 2018-08-31

**Authors:** Paul T. Grogan

**Affiliations:** Stevens Institute of Technology, United States

## Abstract

The experiment studies the effect of technical and social sources of complexity on effort required to complete abstracted design tasks. Parameter design tasks define a set of input design parameters and functional requirements modeled with a linear coupling matrix. Selecting design variables to meet all functional requirements within error limits completes a task. Technical complexity arises from the number and degree of coupling between design parameters. Social complexity arises from the number of designers involved in a task. The experiment includes 10 sessions with between 19 and 24 rounds of randomly generated parameter design tasks each having between two and six design variables and one, two, or three designers. Designers completed individual tasks in parallel during rounds. This article contains raw and post-processed data from 374 completed tasks ranging in effort from a few seconds for simple tasks to more than 15 min for complex ones.

**Specifications table**

**Table:** 

Subject area	*Engineering, Design*
More specific subject area	*Collaborative design processes*
Type of data	*Log files, post-processor, and summary files*
How data was acquired	*Desktop computer*
Data format	*Raw text files, Python post-processor scripts, and post-processed CSV files*
Experimental factors	*Problem Size, Problem Coupling, Team Size*
Experimental features	*Engineering students solving multiple parameter design tasks in the conditions noted above.*
Data source location	*Cambridge, MA*
Data accessibility	*Data is available as a supplementary attachment to this article.*
Related research article	*Grogan, P.T. & de Weck, O.L. Res Eng Des (2016) 27: 221.*https://doi.org/10.1007/s00163-016-0214-7

**Value of the data**

• Parameter design tasks are an important class of abstract engineering design problems for behavioral experiments because of their generalizability and reproducibility.
• This dataset provides a baseline of individual and small group performance on randomly generated fully decoupled and fully coupled tasks for cross-validation of future studies using parameter design tasks.
• Researchers may use this dataset to investigate temporal activities and search strategies within individual tasks to train computational design agents or explain the effectiveness of individual designers or design teams.

## 1 Data

This dataset is included in supplementary data in multiple file formats. The session configuration data (raw/config.json) in JSON format describes details for each session. Key-value pairs in Table 1 include output file names, error tolerance configured in the software application, and any rounds omitted from analysis due to technical problems.

**Table 1:** Session configuration file JSON key-value pairs.

**Key**	**Value**	**Description**
name	String	Label for the session
jsonFile	String	File name for the experiment configuration
logFile	String	File name for the experiment log
errTolerance	Number	Error tolerance describing the maximum distance between design outputs and target functional requirements to yield an acceptable a solution.
omittedRounds	Number[]	List of any round indices omitted from analysis.

The designer data (raw/designers.csv) in comma-delimited CSV format describes demographic information collected from participants. Columns in Table 2 include gender and age.

**Table 2:** Designer demographics file CSV columns.

**Column**	**Value**	**Description**
Designer	Number	Unique designer identifier assigned sequentially by session. For example, session 1 has designers 1, 2, and 3 for zero-based indices 0, 1, and 2 within the session and session 2 has designers 4, 5, and 6.
Gender	String	Designer gender with levels F for female and M for male.
Age	String	Age in years with levels 18–24, 25–29, 30–34, and 35–39.

The experiment configuration data (raw/session#/experiment#.json) in JSON format describes the conditions for experimental rounds. Key-value pairs in Table 3 describe each round with a name, a coupling matrix, a target vector, indices to assign inputs and outputs to designers, and labels for inputs and outputs. Note that rounds with multiple individual tasks completed in parallel are modeled as composite tasks without interaction between designers.

**Table 3:** Experiment configuration file JSON key-value pairs.

**Key**	**Value**	**Description**
name	String	Randomly generated name where Individual designates an individual round and Team designates a team round.
couplingMatrix	Number[][]	Coupling matrix that relates input design parameters to output functional requirements.
targetVector	Number[]	Target vector of design outputs for a zero-error solution.
inputIndices	Number[][]	List of input design variable indices assigned to each designer.
outputIndices	Number[][]	List of output functional requirement indices assigned to each designer.
inputLabels	String[]	List of text labels to display for each input design variable.
outputLabels	String[]	List of text labels to display for each functional requirement.

The experimental log data (raw/session#/session#.log) in comma-delimited text format describes actions taking place during a session. Columns in Table 4 include the timestamp, action type, and action data. Most actions reflect updates to design parameters; however, the log file records the complete state of the inputs and outputs for all tasks at each time.

**Table 4:** Experiment log file fields.

**Column**	**Type**	**Description**
Timestamp	Number	Timestamp of this action in milliseconds since January 1, 1970.
Action type	String	Initialized: task loaded by administrator
		Opened: session loaded by administrator
		Updated: design parameter changed by designer
		Solved: design task completed
Action data	String	Initialized: includes name of task and target output vector
		Opened: name of experiment
		Updated: includes updated design input and output vectors
		Solved: name of task

The post-processor script (post/processor.py) written in the Python language includes a main function to execute the script. The post-processor reads raw data and configuration files to build an in-memory model of a session with the following class hierarchy: PostProcessor composes Session composes Round composes Task composes Action. Each class provides helper functions for post-processing functions.

The post-processor outputs task summary data (post/tasks.csv) in CSV format where each row describes results of a task during a design experiment. Columns in Table 5 include the task session, round, participating designers, task size, degree of coupling, team size, error threshold, and time to complete the task.

**Table 5:** Post-processed task file CSV columns.

**Column**	**Type**	**Description**
Session	Number	The sequential session id containing this task.
Designers	String	Designer id or list of designer ids (delimited by +) in this task.
Round	Number	The sequential round of this task.
Coupled	String	Coupling of this task with levels C for coupled and U for uncoupled.
TaskSize	Number	Number of input design parameters for this task.
TeamSize	Number	Number of designers participating in this task.
Epsilon	Number	Error tolerance allowed for acceptable solutions.
Time	Number	Time to reach an acceptable solution in seconds.

## 2 Experimental design, materials, and methods

### 2.1 Participants and conditions

The study was conducted using an IRB approved protocol. It includes 30 graduate students in technical design-related fields such as aerospace and systems engineering. Participants were assigned to 90-min sessions in cohorts of three based on mutual availability. Sessions were conducted in university classrooms with the three designers and research administrator seated around a single table with computer displays positioned to restrict screen sharing.

The study includes 19 (for sessions 1–3) or 24 (for sessions 4–10) rounds of tasks in partially randomized order to avoid coupled tasks with four variables in first ten rounds. Tasks vary the number of designers, number of design variables, and degree of coupling following the experimental conditions in Table 6. Tasks with one designer are conducted in parallel for all three designers in the same round. A complete description of the experimental design and methods is available in [1] and additional analysis using post-processed data is available in [2].

**Table 6:** Experimental conditions for different task types.

**Designers**	**Variables**	**Coupling**	**Replications**	**Tasks/session**	**Total tasks**	**Omitted**
1	3	Uncoupled	1	3	30	0
1	4	Uncoupled	1	3	30	0
1	6	Uncoupled	1	3	30	3
1	2	Coupled	2	6	60	9
1	3	Coupled	2	6	60	9
1	4	Coupled	2	6	60	19
2	3	Uncoupled	3	3	30	0
2	2	Coupled	3	3	30	0
2	4	Coupled	1	1	10	6
3	3	Uncoupled	2	2	20	0
3	6	Uncoupled	1	1	10	0
3	3	Coupled	1	1	10	0
3	4	Coupled	1	1	10	0

### 2.2 Design task

Design tasks are based on previous work defining a class of randomly generated parameter design tasks for experimental study [3]. This formulation relates a set of design parameters to a set of functional requirements by a square coupling matrix. A task is complete when design parameters achieve a set of target functional requirements within a specified error threshold. Coupled tasks generate coupling matrices by composing orthonormal bases of random vectors from a uniform distribution. Uncoupled tasks generate coupling matrices with diagonal elements randomly selected from − 1 and 1 with equal probability. The target vector is an orthonormal basis of a random vector drawn from a uniform distribution.

Designers work on a task by selecting values of design parameters between upper and lower bounds of 1 and − 1 and observing the effect on functional requirements, attempting to reduce error below the threshold. Multi-actor tasks assign control over design parameters or visibility of functional requirements among multiple designers.

### 2.3 Design interface

**Fig. 1:**
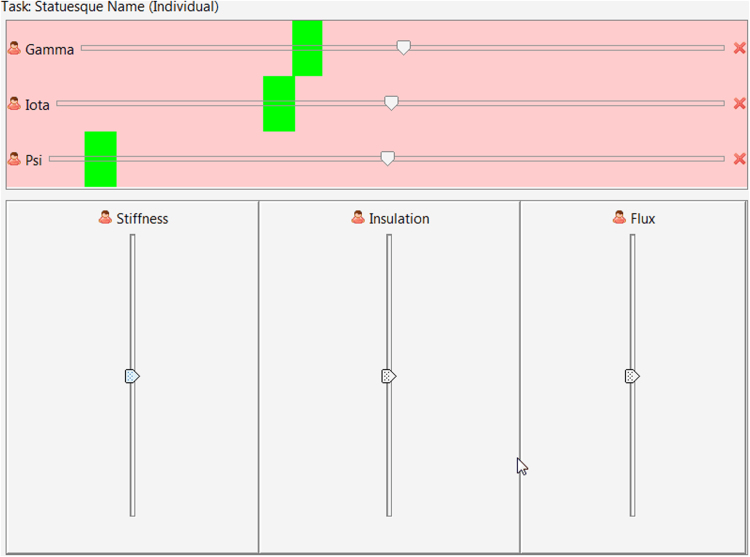
Screen shot of the designer interface with three design parameters and three functional requirements.

Participants interact with a graphical user interface illustrated in Fig. 1 to change design parameters and check error in satisfying functional requirements. Vertical sliders represent design parameters where the thumb displays the current value between upper and lower bounds. Both the mouse (click-and-drag) and keyboard can control design parameters where up and down arrows change by 0.01 units and page-up and page-down keys change by 0.1 units. Horizontal sliders represent functional requirements highlighting the acceptable region for solutions in green where the thumb displays the current value.

A distributed software layer based on the IEEE standard 1516 architecture for simulation interoperability communicates design changes between designer interfaces. A research administrator controls overall task progression from a separate console, which also logs actions. Any change to a design parameter immediately induces an update to show new functional requirements. A chime plays when all tasks are complete in a round, signaling the participants found an acceptable solution.
